# The Associations of Circulating Sphingolipid Levels with Future Loss of Vibration and Light Pressure Sensation in the Lower Limb

**DOI:** 10.3390/biomedicines13122995

**Published:** 2025-12-06

**Authors:** Joshua I. Barzilay, Traci M. Bartz, William T. Longstreth, Elsa S. Strotmeyer, Andrew N. Hoofnagle, David Siscovick, Kenneth J. Mukamal, Rozenn N. Lemaitre

**Affiliations:** 1 Division of Endocrinology, Kaiser Permanente of Georgia, Atlanta, GA 30096, USA; 2 Department of Biostatistics, University of Washington, Seattle, WA 98195, USA; 3 Departments of Neurology and Epidemiology, University of Washington, Seattle, WA 98195, USA; 4 Department of Epidemiology, School of Public Health, University of Pittsburgh, Pittsburgh, PA 15261, USA; 5 Department of Laboratory Medicine and Pathology, University of Washington, Seattle, WA 98195, USA; 6 New York Academy of Medicine, New York, NY 10029, USA; 7 Department of Medicine, Beth Israel Deaconess Medical Center, Brookline, MA 02215, USA; 8 Cardiovascular Health Research Unit, Department of Medicine, University of Washington, Seattle, WA 98101, USA

**Keywords:** sphingolipid, ceramide, peripheral neuropathy, vibration sensation, light pressure sensation

## Abstract

**Background:** Circulating sphingolipids have been implicated in central nervous system degenerative disorders, but their relationship with peripheral neuropathy remains unclear. **Objectives:** To evaluate associations between plasma sphingolipid levels and subsequent loss of vibration and light pressure sensation in the lower limbs of older adults. **Methods:** Plasma concentrations of 11 ceramide (Cer) and sphingomyelin (SM) species were measured in stored samples from 4612 participants in the Cardiovascular Health Study. Vibration sensation was assessed 4–6 years later in 2208 individuals using tuning fork testing, and light pressure sensation was evaluated 11–13 years later in 815 participants using monofilament testing. Sensory impairment was graded on a 3-point scale, with higher scores indicating greater loss. Ordinal logistic regression models examined associations between a doubling of sphingolipid levels and sensory decline, with stratification by diabetes status. **Results:** In primary models, no sphingolipid species showed significant associations with sensory outcomes. However, after adjusting for inflammatory markers, higher SM-16 levels were linked to increased odds of vibration sensation loss (OR 2.08; 95% CI: 1.11–3.90), while higher SM-24 levels were associated with reduced odds (OR 0.68; 95% CI: 0.46–0.998). Significant interactions with diabetes status were observed for light pressure sensation: SM-14 was associated with increased odds of sensory loss in participants with incident diabetes (OR 5.22; 95% CI: 1.58–17.29), and Cer-18 was associated with increased odds in those with prevalent diabetes (OR 2.38; 95% CI: 1.18–4.78). **Conclusions:** Elevated levels of specific ceramide and sphingomyelin species may be predictive of future peripheral sensory loss in older adults, with diabetes status influencing these associations.

## 1. Introduction

Peripheral neuropathy (PN) is a prevalent condition among older adults, with population-based studies indicating that 27–39% exhibit sensory impairments, particularly those with diabetes [1,2]. PN contributes significantly to pain, increased risk of injury and infection, diminished mobility, and elevated mortality rates, independent of diabetes status and traditional cardiovascular risk factors [3].

PN encompasses a group of disorders with multiple etiologies, including metabolic derangements, nutritional insufficiencies, immune-mediated and autoimmune mechanisms, infectious agents, exposure to toxins, and mechanical trauma. Despite diagnostic evaluation, approximately 25% of cases remain idiopathic, underscoring the potential involvement of other pathogenic factors in the development and progression of peripheral nerve dysfunction [4].

Sphingolipids (SLs) constitute approximately 5% of the plasma lipidome and include several hundred species, primarily sphingomyelins (SMs) and a smaller proportion of ceramides (Cers) [5]. These lipids are structural components of cell membranes enabling cells to respond to external stimuli through pathways that regulate apoptosis, inflammation, and stress responses [6]. SL produced inside a cell are incorporated into cell membranes or transported into the circulation via plasma lipoproteins such as apoB or apo-AI [7]. Circulating SLs are synthesized mainly in the liver and their production is influenced by hepatic lipid accumulation, obesity, and insulin resistance [8].

Ceramides are intermediates of SL metabolism which can promote mitochondrial dysfunction, oxidative stress, and activation of pro-inflammatory cytokines impairing cellular energy metabolism [9]. In contrast, SMs–especially those with very-long-chain fatty acids–help maintain membrane integrity by forming platforms (lipid rafts) that stabilize receptor signaling and protect against cellular stress [10]. Dysregulation of these pathways has been implicated in chronic metabolic disorders, cancer, and cardiovascular disease [11,12,13,14].

Our previous research has linked specific SL species—particularly SM-16 and Cer-16, which contain acylated palmitic acid—to increased risk of all-cause dementia in older adults [15]. These species have also been associated with elevated levels of neurofilament light chain, a marker of axonal injury [16]. Additional studies have implicated SLs in neurodegenerative and neuroinflammatory processes, including Alzheimer’s disease, Parkinson’s disease, and psychiatric disorders [17,18,19].

Given the association of SLs with central nervous system disorders, the current study investigates whether circulating plasma SL species are associated with peripheral sensory impairment, specifically vibration and light pressure sensation, in the lower extremities of older adults. We further examine whether these associations are modified by diabetes status.

## 2. Methods

### 2.1. Study Population

**Figure 1:**
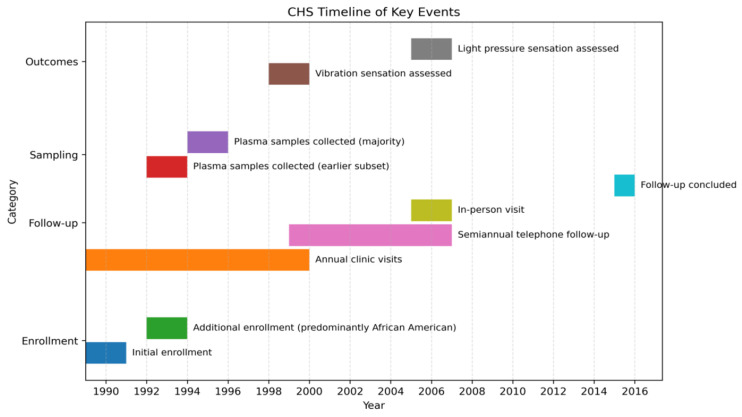
Timeline of Key Cardiovascular Health Study Events.

The Cardiovascular Health Study (CHS) is a prospective, community-based cohort study of adults aged 65 years and older, recruited from Medicare eligibility lists in four U.S. communities [20]. Initial enrollment included 5201 participants between 1989 and 1990, followed by an additional 687 predominantly African American participants in 1992–1993. All participants provided informed consent, and institutional review board approval was obtained at each study site. Annual clinic visits were conducted through 1998–1999, with semiannual telephone follow-up thereafter, including an in-person visit in 2005–2006. A timeline of the events of this study is shown in Figure 1.

### 2.2. Sphingolipids

Plasma samples were collected from 4612 participants—87.3% during the 1994–1995 visit and 12.7% during the 1992–1993 visit—which served as the baseline for this analysis.

Sphingolipid species containing saturated fatty acid chains were quantified using EDTA plasma stored at −70 °C. Lipid extraction and quantification were performed via liquid chromatography–tandem mass spectrometry [14]. Eleven sphingolipid species were measured: five ceramides (Cer-16, -18, -20, -22, -24) and six sphingomyelins (SM-14, -16, -18, -20, -22, -24). SL levels were assayed in 1996–1997 and have been used in prior CHS studies [13,14,15,16] related to heart disease and cognition.

### 2.3. Vibration Sensation Testing

In 1998–1999, foreleg vibration sensation was measured. A handheld 128 Hz tuning fork was struck and held sequentially on the left and right big toes. If no vibration was sensed on either side, the process was repeated over the left and right medial malleoli. If no vibration sensation was detected on either side, the process was repeated over the left and right tibial tuberosities. A participant received a value of 1 if vibration was first detected in either big toe; a value of 2 if vibration was first detected in either malleolus but not below; a value of 3 if vibration was first detected in either tibial tuberosity but not below; and a value of 4 if vibration was not perceived in either tibial tuberosity or below. Vibratory sensation was based on the better of the two lower extremities to mitigate unilateral causes of sensory impairment, such as lumbar radiculopathy.

### 2.4. Pressure Sensation Testing

In 2005–2006, participants were tested for light pressure sensation in the dorsum of the great toe. The response to monofilament testing was classified as: (1) “yes” to 4.17 (1.4 g force) monofilament; (2) “no” to the 4.17 but “yes” to the 5.07 monofilaments; and (3) “no” to both 4.17 and 5.07 monofilaments. “Yes” was defined by feeling the filament in at least 3 of 4 trials with each monofilament.

### 2.5. Covariates

Factors self-reported at the baseline included age, health (excellent or very good vs. worse), any Activity of Daily Living (ADL) difficulty, smoking status (never, former, current), alcohol consumption (>7 drinks per week), and ever diagnosis of cancer. Race, sex, and education (>12th grade) were self-reported at enrollment into the CHS cohort. Weight was measured at the time of SL blood draw and height and waist circumference were measured in 1992–1993. Diabetes status was a three-level variable defined as: never at the time of sensation testing (never); incident after the time of the SL blood draw and before sensation testing (incident); and prevalent at the time of SL blood draw (prevalent). Hypertension was defined as systolic blood pressure ≥140 mmHg or diastolic blood pressure ≥90 mmHg or use of anti-hypertensive medication along with reports of hypertension. Estimated glomerular filtration based on cystatin C levels (eGFRcyst), C reactive protein (CRP), interleukin 6 (IL-6), high-density lipoprotein cholesterol (HDL-C), low-density lipoprotein cholesterol (LDL-C), and triglycerides were measured from blood taken at the baseline visit. A history of coronary heart disease (CHD), myocardial infarction (MI), stroke, or heart failure (HF) was determined at baseline. ApoE4 genotype was measured from blood drawn at enrollment in CHS. Exercise was assessed by response to the question: “During the last week, about how many city blocks or miles did you walk?” Walking is the predominant form of exercise in the age group of the cohort.

### 2.6. Statistical Analyses

Sphingolipid concentrations were log2-transformed to assess the effect of a doubling in levels. Ordinal logistic regression models evaluated associations between sphingolipid levels and sensory impairment. Due to small sample sizes and proportional odds assumption violations, the most impaired vibration categories were combined. Analyses of SM-14, -16, and -18 were adjusted for SM-22; SM-20, -22, and -24 were adjusted for SM-16, with analogous adjustments for ceramides.

Model 1 adjusted for age, sex, race, field center, and year of blood draw.

Model 2 (primary model) added education, smoking, alcohol use, waist circumference, hypertension, diabetes status, height, ApoE4, and eGFRcyst.

Model 3 further adjusted for inflammatory markers (CRP and IL-6).

Sensitivity analyses included inverse probability weighting (IPW) to account for participant attrition and interaction terms between diabetes status and sphingolipid species. The proportional odds assumption was validated for Model 2. Statistical significance was defined as a two-tailed *p*-value < 0.05. No correction for multiple comparisons was applied due to the exploratory nature of the study, so as not to miss a possible significant association. Analyses were conducted using Stata 18.0.

## 3. Results

Among the 2208 participants who underwent vibration sensation testing, 69.2% demonstrated intact sensation at the toes, 18.2% at the ankles but not below, and 12.7% had diminished sensation at or above the tibial tuberosities. For light pressure sensation testing in 815 participants, 57.9% responded to the 4.17 monofilament, 28.7% to the 5.07 monofilament, and 13.4% failed to respond to either.

The distribution of covariates across quartiles of Cer-16 in participants with vibration sensation testing is shown in Table 1. Compared with the other quartiles, the fourth quartile was characterized by the highest waist circumference, systolic blood pressure, triglyceride and CRP levels; the lowest eGFRcyst levels; the least number of blocks walked in the prior week; and the highest prevalences of diabetes and cardiovascular diseases. For the cohort with light pressure sensation testing (Supplemental Table S1) the highest quartile of Cer-16 had the lowest prevalence of self-reported very good or excellent health; the highest prevalence of hypertension; the least number of blocks walked in the prior week; the highest amount of prevalent and incident diabetes; the highest total and LDL-cholesterol and CRP and IL-6 levels; and the lowest eGFR levels.

### 3.1. Vibration Sensation

In primary ordinal logistic regression models, no sphingolipid species were significantly associated with vibration sensation loss (Table 2). However, after adjusting for inflammatory markers (Model 3), higher SM-16 levels were associated with increased odds of vibration loss (OR 2.08; 95% CI: 1.11–3.90), while higher SM-24 levels were linked to reduced odds (OR 0.68; 95% CI: 0.46–0.998).

Sensitivity analyses using inverse probability weighting (IPW) to account for participant attrition revealed a significant association between Cer-16 and vibration loss across all models (Table 3). In Model 3, a doubling of Cer-16 levels was associated with increased odds of vibration impairment (OR 2.14; 95% CI: 1.16–3.93). SM-16 also remained significantly associated with vibration loss in this model (OR 2.46; 95% CI: 1.05–5.76).

No significant interactions were observed between sphingolipid species and diabetes status in relation to vibration sensation loss.

### 3.2. Light Pressure Sensation

In both primary and IPW-adjusted models, no sphingolipid species showed significant associations with light pressure sensation loss (Supplemental Tables S2 and S3). However, significant interactions with diabetes status were identified (Table 4). Among participants with incident diabetes, higher SM-14 levels were associated with increased odds of light pressure sensation loss (OR 5.22; 95% CI: 1.58–17.29). For those with prevalent diabetes, higher Cer-18 levels were associated with increased odds of sensory impairment (OR 2.38; 95% CI: 1.18–4.78).

## 4. Discussion

In this exploratory study of older adults, we examined whether baseline sphingolipid (SL) species predicted future peripheral neuropathy, assessed by vibration and light pressure sensation. In primary models, no SL species were significantly associated with vibration loss. However, after adjusting for inflammatory markers, higher SM-16 levels were linked to increased odds of vibration loss, whereas SM-24 was associated with reduced odds. Sensitivity analyses using inverse probability weighting (IPW), strengthened the SM-16 findings and revealed a significant association between Cer-16 and vibration loss. These results suggest that SM-16 and Cer-16—both derived from palmitic acid—may play a role in sensory loss. Adjustments for CRP and IL-6 amplified these associations, indicating that inflammation may confound or mediate the relationship between these SLs and nerve dysfunction.

For light pressure sensation, no direct associations were observed, even with IPW. However, diabetes status modified the effect of certain SL species: SM-14 was associated with sensory loss in participants with incident diabetes, and Cer-18 in those with prevalent diabetes. These findings, though exploratory, highlight potential metabolic pathways linking SLs, diabetes, and neuropathy. Given multiple comparisons and modest sample sizes, replication in independent cohorts is essential.

The mechanisms relating SLs to PN sensation loss or protection are necessarily speculative in an observational study. Palmitic acid–derived SLs may contribute to neuropathy through pathways involving inflammation, oxidative stress, and mitochondrial dysfunction, all of which impair neuronal energy metabolism [21,22,23,24,25]. Long sensory axons, which convey vibration and light touch sensation, may be sensitive to this disruption. The protective association of SM-24 with vibration sensation may reflect its role in stabilizing membrane integrity and cellular signaling [26,27]. Previous CHS studies have reported similar protective effects of SM species on cognitive decline [15], suggesting shared mechanisms between central and peripheral nervous system health.

Most studies of SL species with nerve function have been done in relation to cognition [15,16,17,18,19]. Only a few studies have examined the association of SL species with diabetic PN, and none with non-diabetic PN. Lopes-Virella et al. investigated the associations between plasma levels of glycosphingolipid species with the presence of symptomatic neuropathy in a type 1 diabetes cohort [28]. Levels of deoxy-C24:0-ceramide, C24:0, and C26:0 ceramide were higher in participants with neuropathy compared to those without neuropathy. In a metabolomic study, Song et al. [29] observed that markers of SL metabolism were related to diabetic PN. Feldman et al. [30] identified 15 metabolomic species, including sphingolipid intermediate product, that differed in diabetic people with and without PN. Other studies have identified phospholipids to be associated with susceptibility to diabetic PN [31,32]. Our findings extend the literature by identifying potential links between SL species and sensory decline independent of diabetes.

Strengths of these analyses include the measurement of SL analytes in a large, well-characterized prospective cohort. The outcomes of vibration and light pressure are complementary to each other and of clinical relevance to older adults. Importantly, the inclusion of participants without diabetes allows for broader generalizability. Limitations of this study include its observational nature, the large number of comparisons, and residual confounding by unmeasured variables, such as vitamin B12 levels. We did not determine when symptoms of PN began, nor did we do electrophysiological studies to detect subclinical nerve dysfunction. The gap of time between obtaining blood samples and sensation testing was long and may have biased the results. Analyses were performed in older adults, and the results may not apply to younger individuals. Finally, we did not have a food frequency questionnaire concurrent with the time of the SL blood draw, thereby preventing assessment of dietary lipid intake (e.g., saturated fatty acids) and its association with circulating SL levels.

## 5. Conclusions

Our findings suggest that specific sphingolipid species may contribute to future peripheral sensory loss in older adults. Associations were strongest for SM-16 and Cer-16 with vibration sensation loss and for SM-14 and Cer-18 in individuals with diabetes. These results highlight potential metabolic pathways underlying PN and warrant further investigation using contemporaneous and repeated measurements to confirm these associations and clarify their clinical implications.

## Figures and Tables

**Table 1:** Baseline descriptive characteristics of participants from the Cardiovascular Health Study with data on vibration sensation by quartile of plasma Ceramide-16 *.

	Q1	Q2	Q3	Q4
Ceramide-16 Levels (μg/mL) Range (n)	0.12–0.22 (555)	0.22–0.25 (547)	0.25–0.29 (554)	0.29–0.80 (552)
BASELINE CHARACTERISTICS				
Age (years)	75.34 (4.32)	75.55 (4.24)	75.49 (4.33)	75.68 (4.66)
Male sex	237 (42.7%)	209 (38.2%)	226 (40.8%)	196 (35.5%)
African American race	101 (18.2%)	85 (15.5%)	65 (11.7%)	73 (13.2%)
Education, at least 12th grade	452 (81.4%)	431 (78.8%)	416 (75.1%)	411 (74.5%)
Any ADL difficulty	75 (13.7%)	69 (12.7%)	63 (11.6%)	80 (14.7%)
Very Good to Excellent self-reported health	241 (43.4%)	223 (40.8%)	199 (35.9%)	174 (31.5%)
Heavy Alcohol Use (>7 drinks/week)	76 (13.7%)	59 (10.8%)	64 (11.6%)	50 (9.1%)
Smoking Status				
Never	256 (46.1%)	279 (51.0%)	260 (46.9%)	277 (50.2%)
Former	257 (46.3%)	230 (42.0%)	241 (43.5%)	229 (41.5%)
Current	42 (7.6%)	38 (6.9%)	53 (9.6%)	46 (8.3%)
Waist Circumference (cms)	96.66 (13.07)	97.14 (11.98)	96.98 (12.04)	98.44 (13.20)
Weight (lbs)	163.35 (31.75)	161.77 (29.31)	160.48 (29.35)	160.62 (32.24)
Height (cms)	165.20 (9.31)	164.36 (9.47)	164.86 (9.08)	164.06 (9.40)
SBP (mm Hg)	132.97 (18.91)	132.11 (18.95)	133.69 (21.46)	134.76 (20.14)
DBP (mmHg)	69.84 (10.21)	69.64 (11.37)	70.14 (11.11)	70.27 (10.97)
Blocks Walked in Prior Week	43.85 (63.80)	42.35 (58.31)	43.36 (62.05)	34.32 (55.18)
PREVALENT DISEASES				
CHD	93 (16.8%)	103 (18.8%)	102 (18.4%)	123 (22.3%)
MI	46 (8.3%)	51 (9.3%)	42 (7.6%)	55 (10.0%)
Stroke	12 (2.2%)	16 (2.9%)	25 (4.5%)	27 (4.9%)
CHF	22 (4.0%)	23 (4.2%)	27 (4.9%)	30 (5.4%)
HTN	310 (55.9%)	294 (53.7%)	317 (57.2%)	311 (56.3%)
Cancer (ever)	106 (19.1%)	113 (20.7%)	93 (16.8%)	111 (20.1%)
Diabetes Status †				
Never	455 (82.0%)	463 (84.6%)	447 (80.7%)	406 (73.6%)
Incident	15 (2.7%)	10 (1.8%)	22 (4.0%)	25 (4.5%)
Prevalent	85 (15.3%)	74 (13.5%)	85 (15.3%)	121 (21.9%)
LABORATORY TESTS				
Total Cholesterol (mg/mL)	194.38 (35.60)	207.32 (33.10)	214.97 (34.89)	224.61 (36.63)
HDL-C (mg/mL)	56.44 (15.36)	54.53 (13.74)	52.08 (13.78)	52.12 (14.12)
LDL-C (mg/mL)	114.89 (31.29)	126.68 (29.84)	133.51 (30.81)	139.73 (33.42)
Triglycerides (mg/mL)	117.03 (67.09)	132.13 (75.01)	151.56 (84.80)	171.92 (98.72)
CRP (mg/L)	4.31 (7.40)	5.02 (8.45)	4.51 (8.15)	6.21 (11.50)
IL-6 (pg/mL)	2.96 (1.89)	3.02 (1.88)	3.12 (2.08)	3.37 (2.13)
eGFRcystatin (mL/min/1.73 m^2^)	72.34 (16.68)	69.93 (15.51)	68.92 (17.19)	67.14 (17.24)
FIELD CENTER				
North Carolina	142 (25.6%)	119 (21.8%)	144 (26.0%)	142 (25.7%)
California	137 (24.7%)	163 (29.8%)	141 (25.5%)	149 (27.0%)
Maryland	105 (18.9%)	118 (21.6%)	126 (22.7%)	145 (26.3%)
Pennsylvania	171 (30.8%)	147 (26.9%)	143 (25.8%)	116 (21.0%)

* Sphingolipid measurement from stored plasma samples drawn from 4612 participants: 4026 (87.3%) from the 1994-1995 visit and 586 (12.7%) from the 1992-1993 visit, which serve as the baseline visits. † Diabetes categories: Never at the time of sensation testing; Incident after the time of the SL blood draw and before sensation testing; and Prevalent at the time of SL blood draw.

**Table 2:** Odds of loss of one level of vibration sensation associated with a doubling of a SL species and *p* values.

Variable	Model 1	Model 2	Model 3
SM-14	1.04 (0.81, 1.33), 0.76	0.97 (0.75, 1.26), 0.83	0.98 (0.74, 1.29), 0.88
SM-16	1.23 (0.69, 2.17), 0.48	1.76 (0.97, 3.18), 0.06	2.08 (1.11, 3.90), 0.02
SM-18	1.16 (0.83, 1.62), 0.39	1.16 (0.82, 1.63), 0.41	1.16 (0.80, 1.67), 0.43
SM-20	0.84 (0.56, 1.26), 0.39	0.76 (0.50, 1.16), 0.20	0.73 (0.47, 1.14), 0.16
SM-22	0.81 (0.54, 1.22), 0.32	0.71 (0.47, 1.09), 0.12	0.66 (0.42, 1.04), 0.07
SM-24	0.73 (0.51, 1.04), 0.08	0.71 (0.49, 1.03), 0.07	0.68 (0.46, 0.998), 0.049
Cer-16	1.31 (0.89, 1.95), 0.17	1.38 (0.92, 2.06), 0.12	1.47 (0.96, 2.26), 0.08
Cer-18	1.15 (0.93, 1.41), 0.19	1.12 (0.91, 1.38), 0.30	1.10 (0.88, 1.38), 0.42
Cer-20	1.19 (0.93, 1.52), 0.16	1.18 (0.92, 1.52), 0.20	1.14 (0.87, 1.49), 0.35
Cer-22	1.07 (0.80, 1.44), 0.65	0.96 (0.71, 1.31), 0.81	0.92 (0.66, 1.28), 0.60
Cer-24	0.90 (0.63, 1.28), 0.55	0.89 (0.62, 1.28), 0.52	0.84 (0.57, 1.24), 0.38

Model 1: Age, Sex, Race, CHS field center, Year of Sphingolipid Measurement. Model 2: Model 1 plus, Education, Smoking Status, Heavy Alcohol Use, Waist Circumference, HTN, Diabetes Status, Height, ApoE4, eGFR (cystatinC). Model 3: Model 2 plus CRP, IL-6. Significant associations are shown in bold. All models: SM-14, -16, and -18 were adjusted for SM-22 and analyses of SM-20, -22, and -24 were adjusted for SM-16, with analogous adjustments for the Cers.

**Table 3:** Odds of loss of one level of vibration sensation associated with a doubling of a SL species using inverse probability weighting to account for participant attrition from the time of blood draw to the time of vibration testing.

SL Species	Model 1	Model 2	Model 3
SM-14	0.95 (0.71, 1.26), 0.70	0.92 (0.69, 1.24), 0.59	0.93 (0.69, 1.26), 0.65
SM-16	1.33 (0.62, 2.86), 0.47	2.01 (0.90, 4.48), 0.09	2.46 (1.05, 5.76), 0.04
SM-18	1.33 (0.82, 2.13), 0.24	1.34 (0.82, 2.19), 0.25	1.32 (0.77, 2.27), 0.31
SM-20	0.95 (0.56, 1.62), 0.85	0.87 (0.51, 1.47), 0.60	0.82 (0.46, 1.44), 0.49
SM-22	0.99 (0.60, 1.63), 0.97	0.86 (0.52, 1.42), 0.54	0.79 (0.46, 1.34), 0.38
SM-24	0.82 (0.53, 1.26), 0.36	0.79 (0.51, 1.23), 0.31	0.76 (0.48, 1.21), 0.24
Cer-16	1.74 (1.01, 3.01), 0.046	1.96 (1.12, 3.43), 0.02	2.14 (1.16, 3.93), 0.01
Cer-18	1.32 (0.97, 1.80), 0.07	1.29 (0.95, 1.75), 0.10	1.26 (0.91, 1.75), 0.17
Cer-20	1.24 (0.89, 1.72), 0.21	1.20 (0.86, 1.67), 0.27	1.12 (0.79, 1.60), 0.52
Cer-22	1.10 (0.77, 1.58), 0.61	0.99 (0.69, 1.44), 0.98	0.92 (0.62, 1.36), 0.66
Cer-24	0.88 (0.59, 1.32), 0.54	0.88 (0.58, 1.34), 0.55	0.80 (0.51, 1.24), 0.32

Model 1: Age, Male sex, African American race, CHS clinic, Year of Sphingolipid Measurement. Model 2: Model 1 plus Education at least 12th grade, Smoking Status, Heavy Alcohol Use, Waist Circumference, HTN, Diabetes status, Height, ApoE4, eGFR (cystatinC). Model 3: Model 2 plus CRP, IL-6. Significant associations are shown in bold. All models: SM-14, -16, and -18 were adjusted for SM-22 and analyses of SM-20, -22, and -24 were adjusted for SM-16, with analogous adjustments for the Cers.

**Table 4:** Odds of loss of one level of light pressure sensation in the great toe associated with a doubling of a sphingolipid (SL) species in diabetes categories * calculated from **model 2** with the interaction of diabetes and SL species added.

SL Species	Significant Interactions of SL Species with Diabetes Status
SM-14	*p* = 0.02
NEVER	0.97 (0.62, 1.52)
PREVALENT	1.06 (0.45, 2.48)
INCIDENT	5.22 (1.58, 17.29)
CER-18	*p* = 0.01
NEVER	0.78 (0.55, 1.12)
PREVALENT	2.38 (1.18, 4.78)
INCIDENT	0.71 (0.25, 2.01)

* Diabetes categories: Never at the time of sensation testing; Incident after the time of the SL blood draw and before sensation testing; and Prevalent at the time of SL blood draw.

## Data Availability

With approved data distribution agreements and institutional review board approval, the data on which this study was based are available from the CHS Coordinating Center at www.chs-nhlbi.org (accessed on 27 November 2025).

## Supplementary Materials

The following supporting information can be downloaded at: https://www.mdpi.com/article/10.3390/biomedicines13122995/s1. Table S1: Characteristics of the Cardiovascular Health Study cohort with light pressure sensation testing by quartile of Ceramide-16 (2005–2006, year 18 of CHS). Table S2: Odds of loss of one level of light pressure sensation in the great toe associated with a doubling of SL species. Table S3: Odds of loss of one level of light pressure sensation associated with a doubling of a SL species using inverse probability.
