# Supplementary material for: The Associations of Circulating Sphingolipid Levels with Future Loss of Vibration and Light Pressure Sensation in the Lower Limb
Source: Biomedicines. 2025 Dec 6;13(12):2995. doi: 10.3390/biomedicines13122995 (PMC12730218; doi:10.3390/biomedicines13122995)
Supplement: Supplementary file 1 [file biomedicines-13-02995-s001.zip › biomedicines-3959700-supplementary.pdf]

Supplementary Table S1*. Characteristics of the Cardiovascular Health Study cohort with light pressure sensation testing by quartile of Ceramide-16 (2005-2006, year 18 of CHS).

	Q1	Q2	Q3	Q4
**Ceramide-16 Range (n)**	0.12-0.22 (207)	0.22-0.25 (231)	0.25-0.29 (201)	0.29-0.54 (176)
**BASELINE CHARACTERISTICS**				
**Age**	74.16 (3.69)	74.22 (3.41)	74.49 (3.69)	74.40 (3.70)
**Male sex**	84 (40.6%)	78 (33.8%)	77 (38.3%)	61 (34.7%)
**African American race**	41 (19.8)	41 (17.7%)	20 (10.0%)	20 (11.4%)
**Education at least 12th grade**	175 (84.5%)	193 (83.5%)	169 (84.1%)	136 (77.3%)
**Any ADL difficulty**	22 (10.7%)	26 (11.3%)	19 (9.5%)	15 (8.6%)
**Very good to Excellent self-reported health**	104 (50.2%)	103 (44.6%)	79 (39.3%)	63 (35.8%)
**Heavy alcohol Use (> 7drinks/week)**	28 (13.5%)	30 (13.0%)	26 (12.9%)	19 (10.8%)
**Smoking status**				
**Never**	101 (48.8%)	130 (56.3%)	99 (49.3%)	88 (50.0%)
**Former**	94 (45.4%)	86 (37.2%)	79 (39.3%)	74 (42.0%)
**Current**	12 (5.8%)	15 (6.5%)	23 (11.4%)	14 (8.0%)
**Waist Circumference**	96.26 (12.67)	96.07 (11.62)	96.54 (11.27)	97.83 (13.98)
**Weight**	164.32 (28.80)	162.13 (27.90)	160.71 (28.43)	161.77 (31.96)
**Height**	165.55 (9.57)	164.23 (8.79)	164.73 (8.92)	164.42 (9.43)
**SBP**	130.55 (18.42)	130.09 (19.08)	130.86 (20.80)	132.61 (19.05)
**DBP**	70.05 (9.94)	70.10 (9.84)	70.52 (11.46)	70.47 (9.81)
**Blocks walked in prior week**	57.83 (74.27)	45.40 (55.73)	49.74 (61.97)	36.60 (50.17)
**PREVALENT DISEASES**				
**CHD**	34 (16.4%)	32 (13.9%)	22 (10.9%)	26 (14.8%)
**MI**	18 (8.7%)	17 (7.4%)	9 (4.5%)	12 (6.8%)
**Stroke**	5 (2.4%)	1 (0.4%)	5 (2.5%)	3 (1.7%)
**CHF**	5 (2.4%)	6 (2.6%)	5 (2.5%)	6 (3.4%)
**HTN**	104 (50.2%)	117 (50.6%)	103 (51.2%)	97 (55.1%)

**Cancer (ever)**	36 (17.4%)	49 (21.2%)	39 (19.4%)	27 (15.3%)
**Diabetes Status†**				
**Never**	165 (79.7%)	190 (82.3%)	160 (79.6%)	125 (71.0%)
**Incident**	12 (5.8%)	16 (6.9%)	17 (8.5%)	21 (11.9%)
**Prevalent**	30 (14.5%)	25 (10.8%)	24 (11.9%)	30 (17.0%)
**LABORATORY TESTS**				
**Total cholesterol**	199.46 (33.72)	210.84 (34.45)	216.36 (30.69)	223.84 (35.48)
**HDL**	56.20 (15.42)	56.07 (14.23)	52.86 (13.06)	51.57 (12.99)
**LDL**	119.71 (30.84)	129.17 (30.21)	134.88 (27.61)	138.73 (32.17)
**Triglycerides**	121.19 (66.86)	128.25 (64.20)	147.44 (87.09)	181.18 (117.15)
**CRP**	3.96 (4.94)	5.10 (9.84)	3.74 (4.42)	5.91 (9.99)
**IL-6**	2.63 (1.46)	2.74 (1.84)	2.82 (2.02)	3.35 (2.06)
**eGFR(cystatinC)**	75.36 (15.90)	73.36 (14.95)	71.44 (16.13)	70.13 (15.94)
**FIELD CENTER**				
**CHS clinic**				
**Winston Salem, North Carolina**	39 (18.8%)	39 (16.9%)	48 (23.9%)	38 (21.6%)
**Sacramento, California**	67 (32.4%)	83 (35.9%)	63 (31.3%)	61 (34.7%)
**Baltimore, Maryland**	35 (16.9%)	42 (18.2%)	35 (17.4%)	34 (19.3%)
**Pittsburgh, Pennsylvania**	66 (31.9%)	67 (29.0%)	55 (27.4%)	43 (24.4%)

* Sphingolipid measurement from stored plasma samples drawn from 4612 participants: 4026 (87.3%) from the 1994-1995 visit and 586 (12.7%) from the 1992-1993 visit, which serve as the baseline visits.

† Diabetes categories: Never at the time of sensation testing; Incident after the time of the SL blood draw and before sensation testing; and Prevalent at the time of SL blood draw.

Supplementary Table S2. Odds of loss of one level of light pressure sensation in the great toe associated with a doubling of SL species.

SL Species	Model 1	Model 2	Model 3
**SM-14**	1.09 (0.75,1.60), 0.65	1.15 (0.77,1.71), 0.49	1.30 (0.85,1.99), 0.23
**SM-16**	1.19 (0.49,2.92), 0.70	1.26 (0.50,3.20), 0.62	1.68 (0.61,4.64), 0.32
**SM-18**	1.32 (0.79,2.21), 0.29	1.25 (0.73,2.15), 0.41	1.56 (0.88,2.79), 0.13
**SM-20**	0.77 (0.41,1.44), 0.41	0.77 (0.40,1.45), 0.42	0.83 (0.42,1.65), 0.59
**SM-22**	0.75 (0.39,1.43), 0.38	0.70 (0.36,1.38), 0.30	0.64 (0.31,1.31), 0.22
**SM-24**	0.72 (0.41,1.26), 0.25	0.70 (0.39,1.25), 0.23	0.63 (0.34,1.17), 0.14
**Cer-16**	1.30 (0.72,2.36), 0.39	1.19 (0.64,2.20), 0.58	1.44 (0.73,2.83), 0.29
**Cer-18**	1.00 (0.74,1.36), 1.00	0.94 (0.68,1.31), 0.73	1.03 (0.72,1.45), 0.89
**Cer-20**	1.00 (0.70,1.42), 0.98	0.98 (0.68,1.41), 0.90	1.16 (0.77,1.73), 0.48
**Cer-22**	1.19 (0.76,1.86), 0.46	1.24 (0.78,1.98), 0.37	1.27 (0.77,2.12), 0.35
**Cer-24**	1.10 (0.63,1.91), 0.74	1.22 (0.69,2.15), 0.49	1.27 (0.69,2.33), 0.44

Model 1: Age, Male sex, African American race, CHS clinic

Model 2: Model 1 plus Education at least 12th grade, Smoking Status, Heavy Alcohol Use, Waist Circumference, HTN, Diabetes Status, Height, ApoE4, eGFR (cystatinC)

Model 3: Model 2 plus CRP, IL-6

All models: SM -14, -16, and -18 were adjusted for SM-22 and analyses of SM -20, -22, and -24 were adjusted for SM-16, with analogous adjustments for the Cers.

Supplementary Table S3. Odds of loss of one level of light pressure sensation associated with a doubling of a SL species using inverse probability weighting to account for participant attrition from the time of blood draw to the time of light pressure testing.

SL Species	Model 1	Model 2	Model 3
**SM-14**	0.72 (0.41,1.29), 0.27	0.82 (0.48,1.40), 0.47	0.86 (0.48,1.54), 0.61
**SM-16**	0.85 (0.25,2.89), 0.79	1.27 (0.38,4.28), 0.70	1.42 (0.37,5.46), 0.61
**SM-18**	1.11 (0.56,2.22), 0.76	1.08 (0.54,2.18), 0.83	1.20 (0.57,2.55), 0.63
**SM-20**	0.61 (0.26,1.45), 0.26	0.45 (0.19,1.10), 0.08	0.51 (0.20,1.32), 0.17
**SM-22**	0.73 (0.28,1.88), 0.51	0.48 (0.18,1.27), 0.14	0.48 (0.17,1.35), 0.16
**SM-24**	0.58 (0.26,1.31), 0.19	0.44 (0.19,1.04), 0.06	0.40 (0.16,1.003), 0.051
**Cer16**	0.71 (0.29,1.74), 0.46	0.96 (0.43,2.13), 0.91	0.97 (0.41,2.31), 0.95
**Cer18**	0.87 (0.57,1.33), 0.52	0.86 (0.56,1.31), 0.47	0.86 (0.54,1.36), 0.51
**Cer20**	1.00 (0.61,1.66), 0.99	0.89 (0.54,1.47), 0.66	1.11 (0.66,1.87), 0.70
**Cer22**	1.12 (0.60,2.11), 0.72	0.95 (0.51,1.76), 0.87	1.10 (0.60,2.05), 0.75
**Cer24**	0.75 (0.31,1.77), 0.51	0.65 (0.29,1.49), 0.31	0.68 (0.29,1.57), 0.37

Model 1: Age, Male sex, African American race, CHS clinic

Model 2: Model 1 plus Education at least 12th grade, Smoking Status, Heavy Alcohol Use, Waist Circumference, HTN, Diabetes Status, Height, ApoE4, eGFR (cystatinC)

Model 3: Model 2 plus CRP, IL-6

All models: SM -14, -16, and -18 were adjusted for SM-22 and analyses of SM -20, -22, and -24 were adjusted for SM-16, with analogous adjustments for the Cers.
